# The impact of caregivers on nosocomial transmission during a COVID-19 outbreak in a community-based hospital in South Korea

**DOI:** 10.1371/journal.pone.0277816

**Published:** 2022-11-21

**Authors:** Hyo-Jin Lee, Hae Kook Lee, Yang Ree Kim

**Affiliations:** 1 Division of Infectious Diseases, Department of Internal Medicine, College of Medicine, The Catholic University of Korea, Seoul, Korea; 2 Department of Psychiatry, College of Medicine, The Catholic University of Korea, Seoul, Korea; Waseda University: Waseda Daigaku, JAPAN

## Abstract

The COVID-19 pandemic becomes a cause of concern for hospital transmission. Caregivers may play an important role as vectors for nosocomial infections; however, infection control for caregivers often is neglected. A nosocomial COVID-19 outbreak occurred in a 768-bed hospital from March 20, 2020, to April 14, 2020. We conducted a retrospective chart review and epidemiologic investigation on all cases. A total of 54 cases of laboratory-confirmed COVID-19 occurred in the community-based hospital. They included 26 (48.1%) patients, 21 (38.9%) caregivers, and 7 (13.0%) healthcare workers. These 21 caregivers cared for 18 patients, and of these, 9 were positive for COVID-19, 6 were negative, and 3 died before testing. Of the 6 negative patients, 3 had no exposure because the caregiver began to show symptoms at least 5 days after their discharge. Of the 9 positive patients, 4 cases of transmission took place from patient to caregiver (one patient transmitted COVID-19 to two caregivers), and 6 cases of transmission occurred from caregiver to patient. Of the 54 hospital-acquired cases, 38 occurred in the 8^th^-floor ward and 8 occurred in the 4^th^-floor ward. The index case of each ward was a caregiver. Counting the number of cases where transmission occurred only between patients and their own caregivers, 9 patients were suspected of having exposure to COVID-19 from their own caregivers. Six patients (66.7%) were infected by COVID-19-confirmed caregivers, and 3 patients were uninfected. Fewer patients among the infected were able to perform independent activities compared to uninfected patients. Not only patients and healthcare workers but also caregivers groups may be vulnerable to COVID-19 and be transmission sources of nosocomial outbreaks. Therefore, infection control programs for caregivers in addition to patients and healthcare workers can be equally important.

## Introduction

While Coronavirus disease 2019 (COVID-19) patients often are infected in their community, nosocomial transmissions are also a great concern to us. Hospitals have substantial proportions of immunocompromised patients, and many admitted patients stay for several days. Furthermore, when a hospital is forced to close due to quarantines, patients’ medical needs cannot be met. Therefore the South Korean government is maintaining strict infection control strategies for COVID-19 in hospitals [1, 2].

The Korean medical system has a shortage of nurses for the number of inpatients. Thus, a caregiver often stays with a patient and performs their basic personal care duties (e.g., walking, toileting, feeding) [3]. Cho et al. reported that 87% of hospitalized patients in Seoul needed caregivers [4]. Patient care is provided by family guardians or occupational caregivers, which are privately employed by the patient, independent of the hospital.

From March 20, 2020, to April 14, 2020, 54 COVID-19 cases occurred in a 768-bed hospital in northeastern Gyeonggi province equipped with regional emergency medical and trauma centers. During this period, many caregivers were infected with COVID-19. They probably had infected their patients, but not all patients with caregivers got infections from their caregivers.

We examined the role of caregivers in nosocomial transmission during a COVID-19 outbreak in a community-based hospital in South Korea.

## Methods

As a university hospital, our hospital has 768 beds and is in northeastern Gyeonggi province, South Korea. The first confirmed COVID-19 case was reported on March 29, 2020, and a total of 54 confirmed cases were reported by April 14, 2020. The outbreak period was set from March 20, 2020, two days before the first confirmed patient showed symptoms to April 14, 2020, the date the last confirmed patient was quarantined. During the outbreak, all healthcare workers (n = 2,072), inpatients (n = 769), and caregivers (n = 133) were tested for COVID-19 (N = 2,974).

COVID-19 testing was performed by real-time reverse-transcription polymerase chain reaction (PCR) using nasopharyngeal and oropharyngeal swabs. Test specimens were collected in accordance with the Korea Disease Control and Prevention Agency guidelines [5]. Nasopharyngeal and oropharyngeal swabs were collected in T-SWAB TRANSPORT™ UTM (Noble Biosciences, Hwaseong, South Korea). RNA was extracted using AdvanSure E3 (LG Chem, Seoul, South Korea). All quantitative real-time PCR amplifications were performed with the PowerCheck™ SARS-CoV-2 Real-Time PCR kit (KogeneBiotech, Seoul, South Korea). SARS-CoV-2 infection was confirmed when *E* and *RdRp* genes of the virus were detected under 35.0 threshold cycles.

All subjects underwent an epidemiologic interview to identify the date of symptom onset; where, when, and how they were exposed to COVID-19; and whether or not they wore personal protective equipment such as a mask. We mapped transmission patterns of all subjects based on the onset of symptoms. In cases where the contact tracing was not clear, possible routes of exposure were investigated. Asymptomatic patients were mapped based on respiratory sample collection date.

COVID-19-positive caregivers and their patients are described in this study. The medical records of patients with infected caregivers were reviewed. The caregiver’s record was referred to as an epidemiological report by epidemiological investigators. Caregivers included full-time occupational caregivers and family caregivers of the patient. Length of nursing days was calculated from the specific caregiver’s nursing start date after 20 March 2020, 2 days before the index case showed symptoms. The Charlson comorbidity index score (CCIS) was calculated using the method described in the previous study [6].

For clinical data, continuous variables are expressed as mean ± standard deviation or median and range according to distribution normality. Categorical variables are expressed as number and proportion. This study was approved by the Institutional Review Board of Uijeongbu St. Mary’s Hospital, The Catholic University of Korea, which provided a waiver of consent because of the retrospective nature of the study (UC20RISI0067).

## Results

### 1) Transmission map

The study site located in northeastern Gyeonggi province was a 768-bed hospital equipped with regional emergency medical and trauma centers. It had 6 ICUs (88 beds) on the 3^rd^-floor and 15 wards from the 2^nd^ to 9^th^ floors. A total of 54 COVID-19 cases were diagnosed in this single community-based hospital from March 20, 2020, to April 14, 2020. These cases included 26 (48.1%) patients, 21 (38.9%) caregivers, and 7 (13.0%) healthcare workers.

**Fig 1:**
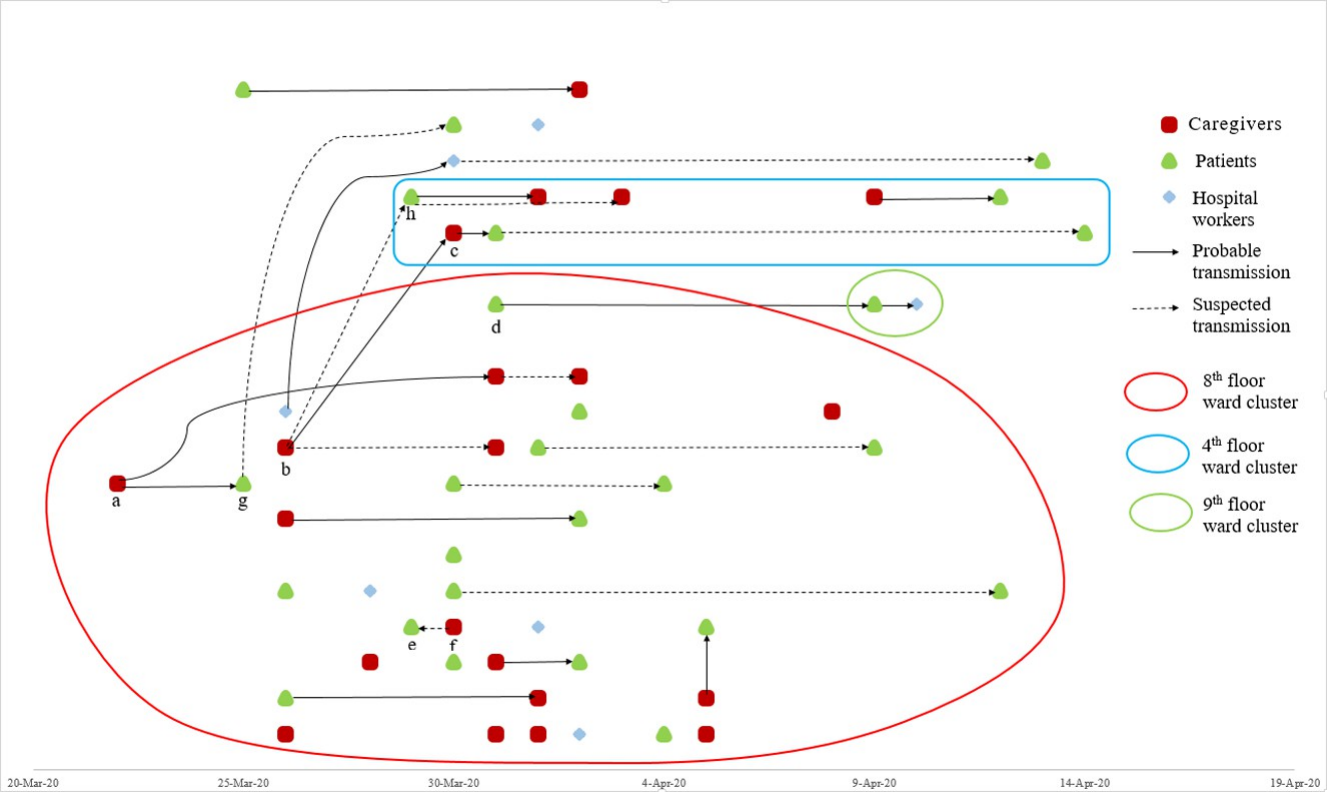
Transmission map.

Fig 1 is a transmission map showing two main clusters and a few sporadic patient infections during the outbreak. Most of the COVID-19-confirmed cases (38/54, 70.4%) occurred in the 8^th^-floor ward. Of these 38 cases, 16 were caregivers, 18 were patients, and 4 were healthcare workers. The 8^th^-floor ward has 20 rooms, 92 beds (4 rooms with 7 beds, 10 rooms with 5 beds, 2 rooms with 4 beds, 2 rooms with 2 beds, and 2 rooms with 1 bed), and 2 nurse stations. One female family caregiver in the 8^th^-floor ward (marked as ‘a’ in Fig 1) developed a fever and respiratory symptoms on March 22, 2020, and was diagnosed with COVID-19 on March 29, 2020 (Fig 1). The patient under the care of that caregiver died before being tested for COVID-19. This caregiver is considered as the index case. While the index case was in the ward, the doors of each adjacent room were ajar slightly, and patients and caregivers often did not wear masks while in the ward. Patients and caregivers did not thoroughly practice hand hygiene. Caregivers shared a pantry and toilet. Most of the healthcare workers were wearing masks well, but sometimes they took off their masks and ate snacks or drank water in the ward. One patient (‘g’ in Fig 1) was not infected by her own caregiver, but by caregiver ‘a’ in the same room. Occupational caregiver ‘b’ in Fig 1 on the 8^th^-floor ward was a friend of caregiver ‘c’, who cared for a patient on the 4^th^-floor; they carpooled to work and frequently visited each other’s wards. A total of 8 COVID-19-confirmed cases occurred in the 4^th^-floor ward: 4 patients and 4 caregivers. Additionally, sporadic cases occurred on the 2^nd^-floor ward (n = 1), 6^th^-floor (n = 2), 7^th^-floor (n = 3), and 9^th^-floor (n = 2).

There were 4 cases of caregivers infected with COVID-19 from patients. Eight patients were transmitted from caregivers.

Transmission map of 26 patients, 21 caregivers, and 7 healthcare workers. There are three transmission chains in the hospital: cluster 1 (red line): patients, caregivers, and healthcare workers in the 8^th^-floor ward (index case ‘a’); cluster 2 (blue line): patients and caregivers in the 4^th^-floor ward (index case ‘b,’ a caregiver on the 8^th^-floor, spread to a caregiver ‘c’ on the 4^th^-floor); cluster 3 (green line): patients and healthcare workers in the 9^th^-floor ward (index case ‘d’ transferred from the 8^th^-floor to the 9^th^-floor before being diagnosed with COVID-19).

Case ‘e’ (patient) showed symptoms earlier than case ‘f’ (caregiver). However, the patient had very little contact with anyone other than the caregiver because she was bedridden and was isolated for 18 days from admission due to pulmonary tuberculosis. Also, fever and respiratory symptoms at the time of hospitalization may have been caused by pulmonary tuberculosis and the time of COVID-19 symptom onset was not clear.

Case ‘g’ (patient) was not exposed to COVID-19 by her own caregiver but by the caregiver ‘a’ of another patient in the same room. Therefore it was excluded from the number of exposed patients by their own COVID-19-confirmed caregivers (Table 2).

Case ‘h’ (patient) was exposed by caregiver ’b’, a friend of ’c’, who was a caregiver for another patient in the same room. Therefore it was excluded from the number of exposed patients by their own COVID-19-confirmed caregivers (Table 2).

Probable transmission indicates the suspected infection transmission route based on contact history.

Suspected transmission indicates when one can’t recollect the contact history, but can assume the transmission route for various reasons.

### 2) Characteristics of caregivers with confirmed COVID-19

A total of 21 caregivers were infected with COVID-19. The mean age (± standard deviation) was 63.7 (± 9.7) years, and only 3 (14.3%) were male. Seven caregivers were asymptomatic at the time of diagnosis. Fourteen caregivers had respiratory symptoms at the time of diagnosis, but their symptoms were mild. The average length of time (± standard deviation) a caregiver cared for a patient before being diagnosed with COVID-19 was 7.2 (± 3.3) days.

There were no significant differences between family and occupational caregiver groups in either case of transmission from patient to caregiver or from caregiver to patient. There also was no significant difference in the period of care between the two groups (Table 1).

**Table 1:** Characteristics of caregivers infected with COVID-19.

	Family caregivers (n = 11)	Occupational caregivers (n = 10)	Total (n = 21)	*P*
**Age in years**	60.6 ± 11.5	67.1 ± 6.2	63.7 ± 9.7	0.131
**Sex, male**	3 (27.3)	0 (0.0)	3 (14.3)	0.246
**Asymptomatic caregiver**	2 (18.2)	5 (50.0)	7 (33.3)	0.280
**COVID-19-positive patient in the care**	5 (45.5)	5 (50.0)	10 (47.6)	0.896
**Infection from patient to caregiver**	2 (18.2)	2 (20.0)	4 (19.0)	1.000
**Infection from caregiver to patient**	3 (27.3)	3 (30.0)	6 (28.6)	1.000
COVID-19-negative patient in the care*	2 (18.2)	4 (40.0)	6 (28.6)	0.534
**Period of care mean (± SD)**	7.6 ± 3.5	6.7 ± 3.1	7.2 ± 3.3	0.526

NOTE. Data are number (%) of patients or mean ± standard deviation (SD) unless otherwise indicated.

COVID-19, coronavirus diseases 2019.

* One caregiver from the family caregiver group and two from the occupational caregiver did not have contact with their patient because their symptoms started at least 5 days after discharge from the hospital.

### 3) Comparisons of infected and non-infected patients exposed to COVID-19 caregivers

**Fig 2:**
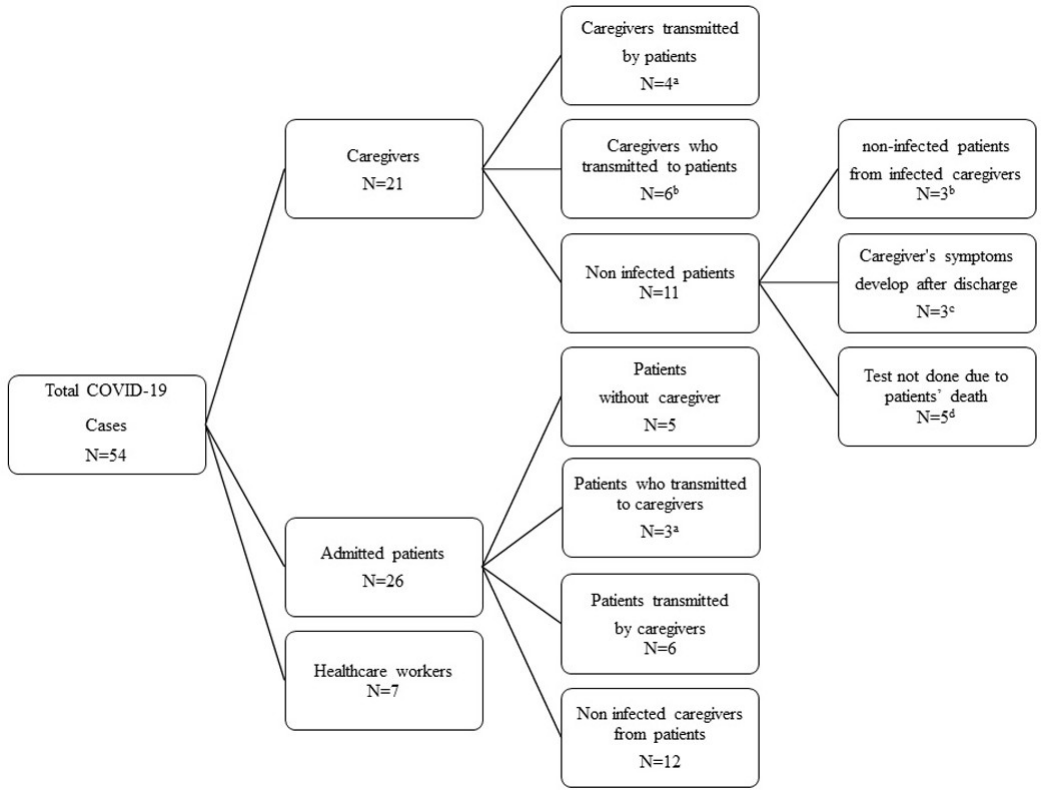
The compositional distribution of 54 COVID-19-confirmed cases between the patients and their own caregivers. ^a^ One patient transmitted COVID-19 to two caregivers. He has one family caregiver and one professional caregiver. ^b^ Six patients infected with COVID-19 from the caregiver and three non-infected patients were compared in Table 2. ^c^ The caregiver’s symptoms developed at least 5 days after discharge and could not be transmitted to the patient. ^d^ Three caregivers were family members of one patient.

Fig 2 shows the compositional distribution of 54 confirmed cases of COVID-19 between the patients and their own caregivers. Counting the number of cases where transmission occurred only between patients and their own caregivers, 21 COVID-19-confirmed caregivers cared for 18 patients (3 family caregivers cared for a single patient and one family caregiver and one professional caregiver cared for the same patient). Of the 18 patients cared for by COVID-19-confirmed caregivers, 9 tested positive for COVID-19 and 6 tested negative. Three patients died before COVID-19 testing, but their causes of death didn’t appear to be linked to COVID-19. Of the 9 positive matched COVID-19 cases for both the patients and their own caregivers, 6 appeared to have been transmitted from caregiver to patient, and 4 were likely to be transmitted from patient to caregiver (one patient transmitted COVID-19 to two caregivers). Of the 6 cases where the caregiver was positive for COVID-19 but the patient was negative, 3 had no COVID-19-related exposure because those caregivers began to show symptoms at least 5 days after discharge. As a result, counting the number of cases where transmission occurred only between patients and their own caregivers, there are nine patients exposed to caregivers infected with COVID-19. Of these, 3 patients were not infected by caregivers and 6 were infected. (In the case of ‘^b^’ in Fig 2).

**Table 2:** Comparisons of infected and uninfected patients exposed to their own COVID-19-positive caregivers.

	Infected patients (n = 6)	Uninfected patients (n = 3)	Total (n = 9)
**Age in years, median (range)**	77 (44-–3)	61 (55–79)	73 (44–83)
**Sex, male**	4 (66.7)	1 (33.3)	5 (55.6)
**Body mass index**	23.9 ± 3.6	27.0 ± 1.8	24.5 ± 3.1
**Smoking**	3 (50.0)	0 (0.0)	4 (36.4)
**Hypertension**	3 (50.0)	1 (33.3)	4 (44.4)
**Diabetes mellitus**	2 (33.3)	2 (66.7)	4 (44.4)
**Length of nursing days**	5.8 ± 3.2	5.3 ± 3.1	6.4 ± 3.4
**Previous ICU admission**	4 (66.7)	0 (0.0)	4 (44.4)
**Previous surgery**	1 (16.7)	0 (0.0)	1 (11.1)
**Self-bathing**	0 (0.0)	1 (33.3)	1 (11.1)
**Self-feeding**	2 (33.3)	2 (66.7)	4 (44.4)
**L-tube feeding**	1 (16.7)	0 (0.0)	1 (11.1)
**Independent walking**	1 (16.7)	1 (33.3)	2 (22.2)
**Alert mental status**	4 (66.7)	3 (100.0)	7 (77.8)
**Bed sores**	1 (16.7)	1 (33.3)	2 (22.2)
**Required suction**	0 (0.0)	0 (0.0)	0 (0.0)
**Indwelling catheter**	6 (100.0)	1 (33.3)	7 (77.8)
**Charlson comorbidity index**	4.7 ± 2.7	7.7 ± 0.6	5.7 ± 2.6
**Caregiver’s age in years, median (range)**	64.5 (47–79)	67 (56–68)	66 (47–79)
**Caregiver’s sex, male**	2 (33.3)	0 (0.0)	2 (22.2)
**Occupational caregiver**	3 (50.0)	2 (66.7)	5 (55.6)
**Asymptomatic caregiver**	2 (33.3)	0 (0.0)	2 (22.2)

**NOTE.** Data are number (%) of patients or mean ± standard deviation (SD) unless otherwise indicated. COVID-19, coronavirus disease 2019; ICU, intensive care unit.

All characteristics in Table 2 were not statistically significant.

Table 2 shows comparisons of 6 infected and 3 non-infected patients exposed to COVID-19-positive caregivers in cases where transmission routes between patients and their own caregivers are considered. Infected patients tended to be older than uninfected patients [77 (44–83) years vs. 61 (55–79) years, median (range)]. Among the infected patients, 66.7% were male, while only 33.3% of uninfected patients were male. Also, 50% of infected patients were smokers, and none of the uninfected patients were smokers. The mean length of care for infected and uninfected patients was 5.8 ± 3.2 days and 5.3 ± 3.1 days, respectively, and there was no significant difference between the two groups. Additionally, 66.7% of infected patients were admitted previously to the intensive care unit, but none of the uninfected patients were. Fewer infected patients were able to perform independent duties (bathing, eating, walking, and maintaining alert mental status) compared to uninfected patients, but the difference was not significant. All uninfected patients had an alert mental status. In contrast, all infected patients had indwelling catheters. The CCIS was higher in uninfected patients than in infected patients.

There was no significant difference in caregiver’s age between the infected and non-infected groups [64.5 (47–79) years vs. 67 (56–68) years] or in the proportion of occupational caregivers and family caregivers (50% vs. 66.7%). At the time of diagnosis, asymptomatic caregivers were 33.3% in the infected patient group and none in the uninfected patient group.

### 4) Enhanced infection control strategy for caregivers after the outbreak

Table 3 shows our hospital’s adapted strategy for caregivers. Prior to this outbreak, the hospital had infection control strategies for patients and healthcare workers, but few specific infection control strategies were in place for caregivers. Infection control strategies for healthcare workers and admitted patients are presented in supplemental information. Occupational caregivers tend to have a different mode of transmission than family caregivers because of the familiar relationships formed during their time working in the hospital. Therefore, the new policy separated occupational caregivers from family caregivers to prevent frequent entry/exit from the hospital. To ensure this behavior, a pledge to abide by hospital policy was written before initiating caregiving, and security service was provided to control entry and exit at the hospital entrance.

**Table 3:** Strategy for caregivers to prevent COVID-19 transmission.

**Common strategies for caregivers**
• Only one caregiver per patient is allowed to enter the ward.
• All caregivers should be examined for SARS-CoV-2 by PCR, up to 48 hours prior to entering the ward.
• Provide a bracelet to caregivers who are authorized to enter the ward.
• A nurse goes around the ward twice a day to check for respiratory symptoms or fever for all caregivers.
• If a caregiver needs to be replaced, the administration team will replace the bracelet and register it in an electronic document.
• Caregivers are not allowed to eat together and should eat alone in designated places.
• It is mandatory for caregivers to wear masks outside the patient room.
• Caregivers are prohibited from entering other patient rooms.
• All caregiver information is digitized and shared with the administration team.
**Strategies for family caregivers**
• Caregivers are prohibited from staying overnight and are allowed to go outside only once a day within a two-hour window.
• The caregiver can enter the hospital without SARS-CoV-2 re-examination only if the patient is admitted to the intensive care unit for one day for postoperative observation. Otherwise, a caregiver who goes out of the hospital must be re-tested for SARS-CoV-2 via PCR before entering the ward again.
**Strategies for occupational caregivers**
• Caregivers should minimize going out of the hospital during their workday.
• One caregiver can care for only one patient at a time.
• Up to 3 patients can be cared for sequentially without leaving the hospital.
• Once the caregiver leaves the hospital after their workday, he or she will have to be re-tested for SARS-CoV-2 via PCR when their new shift begins.

## Discussion

COVID-19 outbreak has become a global pandemic due, in part, to its high infectivity. Of the many outbreaks worldwide, hospital transmissions have had a significant impact on COVID-19 morbidity and mortality. Under the current South Korean medical system, hiring caregivers for admitted patients is common, and they can influence the patients in many ways, including infection transmission [7]. In this study, we examined the transmission links of COVID-19 cases in our hospital, and we demonstrated that caregivers may play an important role in spreading infectious diseases. We also investigated the characteristics of infected and uninfected patients tended by caregivers with confirmed COVID-19.

Among 54 COVID-19 cases, 21 (38.9%) were caregivers. Compared with other groups in the hospital, caregivers may be more vulnerable to COVID-19 exposure. Caregivers often gathered to eat and talk while in close contact with the patients. Also, unlike the patients, they can move around the entire hospital, and medical staff neglected to educate them about infection prevention. Our hospital index case was a caregiver, and the spread between wards was attributed to caregivers. Caregivers seemed to have a greater impact on COVID-19 transmission than other groups.

Asymptomatic infections accounted for 15%–45% of the total number of infections [8, 9]. Several studies have shown that COVID-19 is transmitted from asymptomatic carriers [10, 11]. However, it has been reported that the infectivity transmitted through asymptomatic carriers is lower than that of symptomatic patients, though the viral shedding period is similar [8, 12, 13]. In our study, 33.3% of the caregivers of COVID-19-infected patients were asymptomatic carriers, and none of the caregivers of non-infected patients were asymptomatic carriers. It is important to detect asymptomatic carriers and block transmission.

Counting the number of cases where transmission occurred only between patients and their own caregivers, 6 (66.7%) out of the 9 patients exposed to COVID-19-confirmed caregivers became infected with COVID-19. Several recent studies reported secondary attack rates of 0.7%–30% [14–16]. Meanwhile, additional studies reported that household or family contacts have a higher infection rate than other group contacts [16–18]. Shen et al. reported that COVID-19 spread was much more efficient with close contact than with casual contact (29% vs. 0.6%) [19]. In this study, the rate of infection from caregivers to patients was not significantly different than patient to caregiver. Caregivers had as much contact with patients as with household contacts making them potential external sources of infection. In particular, patients with limited mobility had more contact with their caregivers. Also, sharing a room is a risk factor for infection. A recent study reported a 5.38 odds ratio of COVID-19 infection for sharing a bedroom [14].

Identified risk factors for COVID-19 include age, obesity, and other comorbidities [20–25]. However, sex, diabetes, and hypertension are not risk factors for COVID-19 [24–28]. In our study, infected patients exposed to their own caregivers with COVID-19 appeared more likely to be elderly, male, and smokers than were non-infected patients. Patients who were able to self-bath, self-feed, and walk independently were less likely to be infected than those who were not able to perform those tasks. Hamer et al. reported that physical inactivity (relative risk = 1.32, 95% confidence interval: 1.10–1.58) and smoking (1.42, 1.12–1.79) were associated with COVID-19 infection [29]. It is unclear whether CCIS is a risk factor for COVID-19 infection. Bhatti et al. reported a higher postoperative SARS-CoV-2 infection rate in surgical patients with high age-adjusted CCIS [30]. However, Lio et al. reported that the CCIS in dialysis patients had no significant association with COVID-19 infection [31]. The CCIS was higher in uninfected patients than in infected patients, possibly due to the small number of patients included.

Caregivers were often not considered as a subject of infection control implementation, even though they stayed in the hospital as long as their patients do. However, with an infectious disease that produces asymptomatic spreaders, such as COVID-19, caregivers can be an important transmission factor. Our hospital closed for nearly one month due to this COVID-19 outbreak. After reopening, we began thoroughly managing infections of both patients and caregivers. Caregivers perform nursing duties but often fail to comply with infectious disease prevention guidelines due to their lack of medical knowledge [32]. In the current pandemic era, it is important to carefully manage not only patients and medical staff but also caregivers. Therefore, our hospital has implemented special infection prevention guidelines for caregivers (Table 3).

The effectiveness of universal admission screening for SARS-CoV-2 infections remains controversial [33–35]. Following the COVID-19 outbreak, not only patients but also caregivers began undergoing screening by PCR whenever they entered the hospital. After reopening the hospital, a large outbreak did not occur as before. However, this is not only due to universal screening but also to reduced traffic to and from hospitals through paid examinations. To date, most hospitals in Korea require patients to be tested for COVID-19 before admission.

This study has several limitations. First, it is difficult to show statistical significance because the number of cases is small. Second, because this was a retrospective study, we could not report on caregiver adherence to infection control guidelines such as wearing masks and maintaining good personal hygiene. Third, because the genetic sequencing of SARS-CoV-2 was not conducted during the outbreak period, it didn’t allow us to accurately determine the transmission network.

In conclusion, not only patients and healthcare workers but also caregivers may be vulnerable to COVID-19 and be transmission sources of nosocomial outbreaks. Hospital infection control tends to focus on patients and healthcare workers. In this study, the number of patients or caregivers infected with COVID-19 was higher than that of healthcare workers. Therefore, infection control programs for caregivers in addition to patients and healthcare workers can be equally important.

## Supporting information

S1 File (DOCX)

S1 Dataset (XLSX)
