# Supplementary material for: The impact of caregivers on nosocomial transmission during a COVID-19 outbreak in a community-based hospital in South Korea
Source: PLoS One. 2022 Nov 21;17(11):e0277816. doi: 10.1371/journal.pone.0277816 (PMC9678252; doi:10.1371/journal.pone.0277816)
Supplement: S1 File — (DOCX) [file pone.0277816.s001.docx]

**Supplemental information**

**The Impact of caregivers on nosocomial transmission during a COVID-19 outbreak in a community-based hospital in South Korea**

Hyo Jin Lee^1^, Hae Kook Lee^2^, Yang Ree Kim^1*^

¹Division of Infectious Diseases, Department of Internal Medicine, College of Medicine, The Catholic University of Korea, Seoul, Korea

^2^Department of Psychiatry, College of Medicine, The Catholic University of Korea, Seoul, Korea

**Correspondence to:** Yang Ree Kim M.D., Ph.D.

E-mail: [yrkim@catholic.ac.kr](mailto:yrkim@catholic.ac.kr) (YRK)

**Supplemental table 1.** **Strategy for healthcare workers to prevent COVID-19 transmission**

| - All healthcare workers should adhere to rigorous hand hygiene practices in accordance with WHO guidelines. |
| --- |
| - All healthcare workers should be monitored daily for fever or respiratory symptoms. |
| - All healthcare workers should immediately report any epidemiological link with COVID-19 to the head of the department. Then the department head should report to the Infection Control Office and follows the measures of the Infection Control Office. |
| - All healthcare workers should report any suspicious symptoms such as fever or respiratory symptoms to the head of the department immediately. Then the healthcare workers must be tested for SARS-CoV-2 by PCR and will be excluded from work until they test negative for COVID-19. |
| - All healthcare workers are required to follow the proper use of face masks to cover their noses and mouths while working in the hospital. |
| - All healthcare workers should wear face shields when performing procedures that may generate aerosols in all patients. |
| - All healthcare workers are required to refrain from eating and drinking outside designated areas. |
| - All healthcare workers are recommended to ventilate the workplace every morning, noon, and evening. |
| - All healthcare workers are recommended to clean and disinfect high-touch surfaces three times a day. |
| - All healthcare workers are recommended to minimize the number of attendees and shorten meetings in the hospital. |

**Supplemental table 2.** **Strategy for admitted patients to prevent COVID-19 transmission**

| - All patients should be examined for SARS-CoV-2 by PCR, up to 48 hours prior to entering the ward. |
| --- |
| - All patients should be monitored for fever or respiratory symptoms at least three times per day. |
| - All patients should immediately report any epidemiological link with COVID-19 to the nurse in charge. Then the nurse should report to the Infection Control Office and follows the measures of the Infection Control Office. |
| - If there is a patient with suspected COVID-19 symptoms, medical staff should perform SARS-CoV-2 by PCR on the patient and consult the infectious disease department. |
| - All patients are required to wear a mask during hospitalization, except for patients who cannot wear a mask for medical reasons. |
| - Inpatient ward transfers should be minimized. |
| - Patients are prohibited from entering other patient rooms. |
| - Patients are prohibited from staying overnight and are allowed to go outside only once a day within a two-hour window. |
| - Patients room doors should be kept closed. |
